# Internet Survey of Awareness and Behavior Related to HPV Vaccination in Japan

**DOI:** 10.3390/vaccines9020087

**Published:** 2021-01-25

**Authors:** Risa Kudo, Masayuki Sekine, Manako Yamaguchi, Megumi Hara, Sharon J. B. Hanley, Yutaka Ueda, Asami Yagi, Sosuke Adachi, Megumi Kurosawa, Etsuko Miyagi, Takayuki Enomoto

**Affiliations:** 1 Department of Obstetrics and Gynecology, Niigata University Graduate School of Medical and Dental Sciences, Niigata 951-8510, Japan; pearpear@med.niigata-u.ac.jp (R.K.); manako0131@med.niigata-u.ac.jp (M.Y.); sadachi@med.niigata-u.ac.jp (S.A.); m-kurosawa@med.niigata-u.ac.jp (M.K.); enomoto@med.niigata-u.ac.jp (T.E.); 2 Department of Preventive Medicine, Faculty of Medicine, Saga University, Saga 849-8501, Japan; harameg@cc.saga-u.ac.jp; 3 Department of Obstetrics and Gynecology, Hokkaido University Graduate School of Medicine, Sapporo 060-8638, Japan; sjbh1810@med.hokudai.ac.jp; 4 Department of Obstetrics and Gynecology, Osaka University Graduate School of Medicine, Suita 565-0871, Japan; y.ueda@gyne.med.osaka-u.ac.jp (Y.U.); a.yagi@gyne.med.osaka-u.ac.jp (A.Y.); 5 Department of Obstetrics and Gynecology, Yokohama City University School of Medicine, Yokohama 236-0004, Japan; emiyagi@yokohama-cu.ac.jp

**Keywords:** HPV vaccination, cervical cancer, Internet survey, sexual behavior, cancer screening

## Abstract

Recommendations for HPV vaccines were suspended in 2013 due to unfounded safety fears in Japan. We aimed to clarify the differences between vaccinated and unvaccinated females in their awareness, knowledge, and behaviors toward cervical cancer, HPV vaccination and sex. Questionnaires were administered online to women aged 16 to 20. We conducted investigations for the following: awareness, knowledge, and actions for cervical cancer, HPV vaccination, and sexual activity, as well as items related to participants’ social background. The survey in 828 girls revealed three points. The first is that more than half of the surveyed Japanese girls had poor knowledge about cervical cancer screening, HPV, or HPV vaccines. The second is that those in the unvaccinated group had a particularly poor knowledge of the subject and tended to have higher sexual activity. The final is that only 0.5% of the girls experienced changes in awareness about sexual activity after vaccination. In conclusion, this is the first large-scale survey analyzing the association between HPV vaccination and sexual activity in Japanese girls. Not only do unvaccinated girls not benefit from vaccines, but they also tend to engage in high-risk sexual behavior, and thus it is even more important to provide information on the effectiveness of vaccines and the usefulness of cancer screening.

## 1. Introduction

The bivalent vaccine and quadrivalent vaccine were approved as human papillomavirus (HPV) vaccines for primary prevention against cervical cancer in Japan in 2009 and 2011, respectively [1]. Public funding for HPV vaccinations for women aged 13–16 years was initiated in 2010 by municipalities and in April 2013 it was included in the national immunization program (NIP) for women aged 12–16 years. HPV vaccination rates in Japan are high (70%) despite the vaccine not being administered within school-based programs, but individually in clinics [2,3]. However, around the same time, there was sensationalized reporting on the various symptoms which were misunderstood to occur following immunization. This prompted the Ministry of Health, Labor, and Welfare to suspend proactive recommendations for the HPV vaccine in June 2013 [1]. Vaccination rates dropped sharply to <1% in less than a year [2]. To this day, the vaccination rate continues to be close to zero despite the HPV vaccine still being included in the NIP and women within the target age range being able to receive HPV vaccination for free [3,4].

HPV vaccination is recommended as a national project in many countries around the world. Regarding the effectiveness of HPV vaccines, many papers reported showing the preventive effect on the HPV infection and precancerous lesions [1,5,6,7,8], and, in addition, a paper showing the effect of reducing invasive cervical cancer was published by Sweden in October 2020 for the first time in the world. HPV vaccination has been shown to reduce the risk of invasive cervical cancer by 63%, and vaccination under the age of 17 years reduces the risk by 88% [9]. A Cochrane review that analyzed safety of the HPV vaccine reported that HPV vaccination increased swelling at the site of injection, but did not increase serious adverse reactions [10]. The WHO continuously evaluates the latest data on HPV vaccines worldwide and reports that no safety issues have been found that would require changes to HPV vaccine recommendations [11]. They conclude that all bivalent, tetravalent, and 9-valent vaccines show excellent safety and efficacy data, and each country has published a position paper on HPV vaccination and a guideline on cervical cancer screening [12,13].

Cervical cancer is on the rise in Japan in terms of both morbidity and mortality. The increasing morbidity rate, particularly among young women of reproductive age, has been considered problematic alongside late births [14]. Rates of cervical cancer screening as secondary prevention in Western countries are 60–80%, whereas those in Japan are 30–40%, with rates among those in their early 20 s in particular at 10% or less [15]. As the only country with increasing rates of cervical cancer, Japan is behind the rest of the world.

It is anticipated that seven years after suspension of proactive recommendations, simply reinstating proactive recommendations at the national level will not sufficiently recover vaccination rates that have rapidly fallen to almost zero. Other than the concern for adverse events, people opposed to resuming HPV vaccination recommendations argue that girls vaccinated against HPV would become more sexually active and less willing to undergo cervical cancer screening, eventually leading to no desirable reduction in cervical cancer morbidity risks [16].

This study aimed to clarify the differences between vaccinated and unvaccinated females in their awareness, knowledge, and behaviors toward cervical cancer, cervical cancer screening, HPV vaccination, and sex and investigated methods for recovering the drastically reduced vaccination rate by analyzing changes after HPV vaccination.

## 2. Materials and Methods

### 2.1. Internet Survey

Questionnaires were administered online to women aged 16 to 20 across Japan (i.e., those who belonged to a generation where publicly funded HPV inoculations have been conducted) from 5 February 2015 to 12 February 2015. Questionnaires were emailed to target-age women who were registered as monitoring members on Macromill. Basic attributes—such as sex, age, occupation, place of residence, and marital and child status—were registered for monitoring members on Macromill. In this study, we used vaccination and willingness to answer questionnaires as allocation factors and selected enrollees so that the age composition of the vaccinated and unvaccinated groups would be equal. We conducted investigations for the following in both vaccinated and unvaccinated groups: awareness, knowledge, and actions for cervical cancer, cervical cancer screening, HPV vaccination, and sexual activity, as well as items related to participants’ social background such as socioeconomic status, drinking history, and smoking history.

### 2.2. Statistical Analysis

The data were analyzed using SPSS (Ver25) (IBM, Chicago, IL, USA). Categorical data are presented as absolute numbers and percentages. Continuous data are presented as the means ± standard deviation. Pearson’s chi-squared test, Fisher’s exact test, Student’s t-test, the Mann–Whitney test, logistic regression analysis were used to check for differences in baseline characteristics between the vaccinated and unvaccinated populations. Univariate and multivariate logistic regression analyses were conducted to assess awareness and knowledge for HPV vaccine, cervical cancer, and cervical cancer screening. Statistical analysis of knowledge was evaluated using logistic regression analysis. The three answers, “savvy”, “vague”, and “none”, were evaluated as 2, 1, and 0 points, respectively, and scaled. The odds ratio in the vaccinated group was calculated by univariate analysis with the unvaccinated group as the reference level. After that, multivariate analysis was performed on the items for which a significant difference was found in the univariate analysis and the items with strong correlation were evaluated. A *p*-value of < 0.05 was considered statistically significant. The questionnaire study was conducted anonymously. The subjects were fully informed of the aim of this study. The answers who gave consent filled out the questionnaire. The study was approved by the Ethics Review Board of Niigata University Graduate School of Medical and Dental Sciences (Approval Number 2015-1866).

## 3. Results

### 3.1. Background of Participants

We received questionnaire responses from 828 people. There were 414 people each for the vaccinated and unvaccinated groups. The age distribution was as follows: 16 years, 86 people (10.4%); 17 years, 132 (15.9%); 18 years, 228 (27.5%); 19 years, 274 (33.1%); and 20 years, 108 (13.0%) (Table 1). The survey was conducted so as not to be biased for different parts of Japan, and there were no significant differences regarding the place of residence in the vaccinated and unvaccinated groups. Marriage was significantly more common in the unvaccinated group compared to the vaccinated group, with 32 (7.7%) versus 8 people (1.9%), respectively (*p* < 0.001). There were no significant differences in sexual experience between the vaccinated and unvaccinated groups, with 135 (32.6%) versus 148 (35.7%) people, respectively (*p* = 0.39). The percentage of students was significantly higher in the vaccinated group compared to the unvaccinated group, with 390 (94.2%) people versus 359 (86.7%), respectively (*p* < 0.001). As for life history, smoking history was significantly more frequent in the unvaccinated group (*p* = 0.014). Among the participants who were registered as students, over 60% responded with “unknown” for their annual family income, and no significant differences were found between the vaccinated and unvaccinated groups (*p* = 0.108). To gauge economic status, participants were asked whether they had attended cram schools; attendance was significantly higher in the vaccinated group compared to the unvaccinated group, with 276 people (66.7%) versus 238 (57.5%), respectively (*p* = 0.001).

### 3.2. Knowledge of Cervical Cancer Screening and HPV Vaccination

Two items for cervical cancer screening, two for cervical cancer, two for HPV, and one for the effects of HPV vaccination are shown (Table 2, Supplemental Table S1). Of these, the vaccinated group had significantly more knowledge of the following items: “Necessity of regular cancer screening” (odds ratio (OR) = 1.29, 95% confidence interval (CI): 1.08 to 1.55, *p* = 0.005), “Cervical cancer negatively affects pregnancy” (OR = 1.33, 95% CI: 1.11 to 1.60, *p* = 0.002), “The virus leading to cervical cancer infects during sexual intercourse” (OR = 1.19, 95% CI: 1.00 to 1.42, *p* = 0.048), and “HPV vaccination is effective for cervical cancer prevention” (OR = 2.03, 95% CI: 1.11 to 1.60, *p* < 0.001). The most relevant knowledge of HPV vaccination was the effectiveness of the HPV vaccine. Only two items relating to cervical cancer were known by over 50% of participants, those items were “Cervical cancer negatively affects pregnancy” and “Cervical cancer may cause death.” It was noteworthy that only 8.9% of the unvaccinated knew that “HPV vaccination is effective for cervical cancer prevention.”

### 3.3. Sexual Activity and Behavior

Analyses of the items related to sexual debut, sexual partners, and sexually transmitted infections were restricted to experienced women (*n* = 283). Analyses of condom use and pregnancy were restricted to unmarried and experienced women (*n* = 247) (Table 3). Age at sexual debut was significantly lower in the unvaccinated group compared to the vaccinated group, with 15.8 (± 2.4) years versus 16.6 (± 2.2) years, respectively (*p* = 0.008). The number of experienced individuals was also significantly higher in the unvaccinated group compared to the vaccinated group, with 7.0 (± 12.4) versus 4.0 (± 6.2), respectively (*p* = 0.016). Among all the 828 individuals, 232 (28.0%) reported meeting men via the Internet, with significantly (*p* = 0.0032) more women in the unvaccinated group (*n* = 134; 32.4%) than in the vaccinated group (*n* = 98; 23.7%) having done so. The significant difference was identified for condom use, though there were more women in the vaccinated group who said they used a condom “every time” or “frequently” (83.7%, *p* = 0.046). Pregnancy was significantly more prevalent in the unvaccinated group compared to the vaccinated group, with 14 people (11.9%) versus 6 (4.7%), respectively (*p* = 0.036). In terms of history of sexually transmitted infections, no significant differences were observed (*p* = 0.72).

### 3.4. Change in Awareness of Sexual Activity after HPV Vaccination

**Figure 1:**
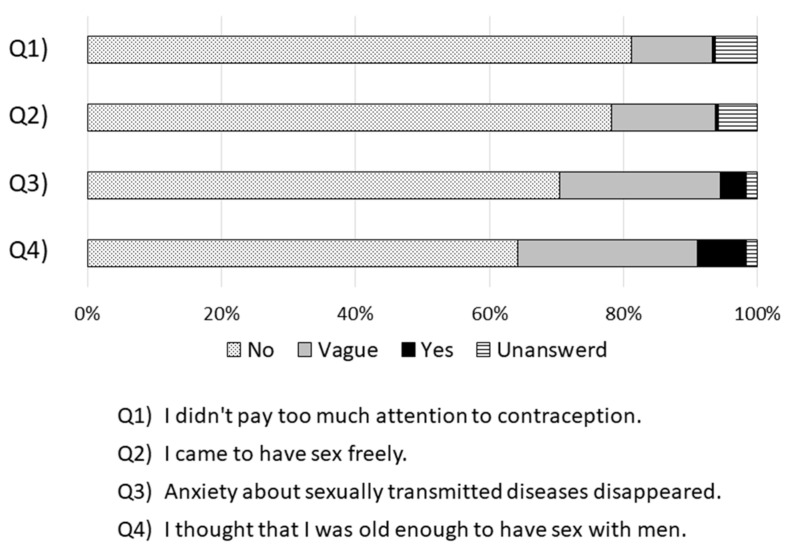
Change in awareness of sexual activity after HPV vaccination.

We asked about changes in awareness and actions regarding contraception, free sexual behavior, and sexually transmitted diseases after inoculation (Figure 1). Results showed that those who answered “I didn’t pay too much attention to contraception” or “I came to have sex freely” after vaccination were extremely few (*n* = 2; 0.5%). There were 16 (3.9%) women who answered “Anxiety about sexually transmitted diseases disappeared,” 30 (7.2%) who answered “I thought that I was old enough to have sex with men,” and over 90% of women who did not observe any obvious changes in awareness of sexual activity following vaccination.

Over 90% of women did not observe any obvious changes in awareness of sexual activity following vaccination. Those who answered “I didn’t pay too much attention to contraception” or “I came to have sex freely” after vaccination were extremely few (n = 2; 0.5%).

### 3.5. Anxiety and Preventive Action against Cervical Cancer

Results for anxiety about cervical cancer and sexually transmitted diseases and preventive action against cervical cancer are shown in Table 4. No significant differences were observed between the vaccinated and unvaccinated groups for anxiety associated with cervical cancer (*p* = 0.48) or sexually transmitted diseases (*p* = 0.15). No significant differences were observed between the two groups regarding intent to regularly undergo cervical cancer screening (*p* = 0.62), but the percentage of those who agreed with the statement “Vaccination is preferable if it can prevent cancer” was significantly higher in the vaccinated group compared to the unvaccinated group (*p* < 0.001).

## 4. Discussion

This survey focused not only on comparisons between the groups of women who had and had not received a prior HPV vaccination, but also on the respective changes in awareness and behavior towards cervical cancer prevention and sexual behavior. The survey revealed three points. The first one is that young Japanese women have poor knowledge about cervical cancer and HPV. The second is that those in the unvaccinated group have a particularly poor knowledge of the subject and tend to have higher sexual activity. The final one is that only a minority of women experienced changes in awareness about sex and developed sexual activity after vaccination. Such a large-scale survey has not been previously reported in Japan and is the first report in Japan analyzing the association between HPV vaccination and sexual activity in girls.

Some opponents of the recommendations for HPV vaccination argue that increased sexual activity of the girls who have received HPV vaccination and their failure to seek cervical cancer screening care may conversely increase the risk of cervical cancer [16]. There have been international reports that parents are concerned about the effects of HPV vaccination on sexual activity in children [17], while others have reported that HPV vaccination does not affect sexual activity [18,19,20]. In Japan, there have been no previous report which investigated how awareness about sex and sexual activity changed after HPV vaccination. The current study showed no apparent increase in “changes in sexual activity” among the girls who received HPV vaccination such as altered awareness of sex and increased risks of HPV infection. Only two (0.5%) respondents said that they became sexually active and no longer worried about contraception after vaccination, and the majority responded that they did not change their sexual behavior after vaccination. The results did not show that those in the vaccinated group had riskier sexual behaviors compared to those in the unvaccinated group. Instead, it was the unvaccinated group that tended to have riskier sexual behavior. The unvaccinated group was recognized as a high-risk group for cervical cancer not only because they were not vaccinated but also because of their sexual behavior. Correlations with socioeconomic status have been reported as factors associated with higher sexual risks [21,22]. It is difficult to assess socioeconomic status using an Internet survey for teenage women; this survey also found it difficult to obtain information on family income (unknown: 66%). We were able to clarify socioeconomic differences between the groups by using the attendance of cram schools as a surrogate measure. Several studies have shown that sexual activity and education were correlated [23,24,25]; these findings support our results.

As for awareness of cervical cancer screening, there were significant differences between the vaccinated and unvaccinated groups for anxiety towards cervical cancer and intention to regularly undergo cervical cancer screening; the results did not indicate any negative impact of HPV vaccination on the intent to receive cervical cancer screening. According to the correlations between HPV vaccination and intention to undergo cervical cancer screening reported in Japan, more HPV-vaccinated females tend to undergo cervical cancer screening than unvaccinated women [26]. As such, promoting HPV vaccination, which is a form of primary prevention, in combination with increasing rates of cervical cancer screening, which is a form of secondary prevention, is crucial in eliminating cervical cancer.

Sex education in Japan grew alongside the mounting anxiety about AIDS in the 1980s, and course guidelines were revised in 1992 to continue the education process from elementary school to high school [27]. In 2002, however, a Diet member noted the description of oral contraceptives written in the accessory teaching materials on sex education and criticized the “remarkable absence of perspectives on the sanctity of sexual intercourse in fostering life,” resulting in a severe pushback on sex education across Japan and serious reductions in sex education content in course guidelines in 2004–2018. Afterwards, there were increased concerns that sex education would increase sexual awareness in children and decrease the age at which sexual behavior would start; learning guidelines with reduced content are still used today [28,29]. The United Nations Educational, Scientific, and Cultural Organization (UNESCO) collaborated with the World Health Organization to conduct a worldwide survey of sex education and concluded that comprehensive sex education would not hasten sexual behavior, but in fact, bring about more prudent sexual behavior. As a result, an International Sexuality Education Guidance was developed by UNESCO in 2009 to provide effective sex education, and this has established a global institutional basis for sex education [30].

In our survey, young women were asked about their knowledge of cervical cancer and HPV, and their levels of familiarity were lower than expected. The vaccinated group tended to have more knowledge of cervical cancer and HPV, but “Cervical cancer negatively affects pregnancy” was the only statement which more than half of the participants reported being “familiar” with. This were thought to be caused by problems in sex education guidance in schools—as mentioned above—and inadequate information regarding cervical cancer and its causes provided by attending physicians during vaccinations. It is presumed that girls who are vaccinated do so without knowing what they are being vaccinated for despite HPV vaccination being individualized by health facilities. This may be because approximately 80% of the vaccinated respondents were inoculated in medical and pediatric departments that do not treat cervical cancer. In the United Kingdom, parents or individuals are required to be fully informed before vaccination; vaccinating healthcare professionals must know about cervical cancer even for school-based inoculations. Similarly, Japan must provide accurate medical information not only to patients, but also to medical workers and, in its quest to reinitiate proactive recommendations, create a system that sufficiently provides explanations during vaccination.

There are three primary limitations to this survey. The first is that vaccination history was self-reported and may have been misclassified. Matching the archived immunization records showed a low self-reported negative predictive value of approximately 50%; of those who responded that they did not receive HPV vaccination, 11% of girls were vaccinated (i.e., they forgot about their three inoculations) [31]. In other words, there are limits to the accuracy of inoculation information in the questionnaire survey. Inoculation records in Japan are managed by approximately 1700 municipalities, and the lack of integration at the national level makes the inoculation records difficult to examine. This issue is a major challenge for vaccine administration in Japan for accurately grasping the adverse reactions that might occur after HPV vaccination and advancing discussions toward reinitiating proactive recommendations for HPV vaccination and NIP introduction of nine-valent vaccines. The second is that sexual activity could not be accurately assessed because of the considerable number of married individuals in the unvaccinated group. As a countermeasure, we believe that some biases could be eliminated by conducting analyses on contraception and pregnancy only among unmarried individuals. The third is that the survey targeted women aged 16–20 years who received vaccination at public expense at that time. Since this age is not target for cervical cancer screening, there may be a tendency for women of this age to be less interested in cervical cancer and cervical cancer screening.

Japan is a monoethnic nation, and Japanese people follow national policies and behave similarly to those around them [32]. It is important to disseminate and provide accurate data on the efficacy and adverse reactions of vaccines to parents, schools, and municipalities to re-disseminate HPV vaccination in Japan in the future. Society must also be informed that alterations in awareness and behavior—such as increased sexual activity and loss of cervical cancer screening—rarely occur among vaccinated girls.

## 5. Conclusions

In conclusion, not only do unvaccinated individuals not benefit from vaccines, but they also tend to engage in high-risk sexual behavior, and thus it is even more important to provide information on the effectiveness of vaccines and the usefulness of cancer screening in these groups. Measures such as improving sex education in compulsory education, providing accurate information on cervical cancer and HPV vaccination for vaccinated girls by physicians in charge of vaccination, and creating materials and systems for them are needed to reinitiate positive recommendations for subsequent dissemination of HPV vaccination.

## Figures and Tables

**Table 1:** Background of participants in this Internet survey.

	Vaccinated (*n* = 414)		Unvaccinated (*n* = 414)		*p*-Value
**Age (years)**					
16	43	(10.40%)	43	(10.40%)	1.00 *
17	66	(15.90%)	66	(15.90%)	
18	114	(27.50%)	114	(27.50%)	
19	137	(33.10%)	137	(33.10%)	
20	54	(13.00%)	54	(13.00%)	
**Marital status**					
married	8	(1.90%)	32	(7.70%)	<0.001 *
unmarried	406	(98.10%)	382	(92.30%)	
**Sexual experience**					
yes	135	(32.60%)	148	(35.70%)	0.39 *
no	255	(61.60%)	246	(59.40%)	
unanswered	24	(5.80%)	20	(4.80%)	
**Occupation**					
student	390	(94.20%)	359	(86.70%)	<0.001 *
employed	16	(3.90%)	25	(6.00%)	
housewife	8	(1.90%)	30	(7.20%)	
**Smorking history**					
yes	30	(7.20%)	51	(12.30%)	0.014 *
no	384	(92.80%)	363	(87.70%)	
**Family income (JPY)**					
<2 milion	41	(9.90%)	44	(10.60%)	0.108 ^†^
2–4 milion	30	(7.20%)	45	(10.90%)	
4–6 milion	22	(5.30%)	22	(5.30%)	
6–10 milion	32	(7.70%)	30	(7.20%)	
10 milion<	12	(2.90%)	4	(1.00%)	
unknown	277	(66.90%)	269	(65.00%)	
**Economic circumstances (self-judgment)**					
very good	70	(16.90%)	57	(13.80%)	0.039 ^‡^
good	112	(27.10%)	107	(25.80%)	
normal	158	(38.20%)	160	(38.60%)	
poor	64	(15.50%)	65	(15.70%)	
very poor	10	(2.40%)	25	(6.00%)	
**Attending cram school**					
yes	276	(66.70%)	238	(57.50%)	0.001 *
no	138	(33.30%)	168	(40.60%)	
unknown	0	0	8	(1.90%)	

* Chi-squared test; ^†^ *t*-test (exclude “unknown”); ^‡^ *t*-test.

**Table 2:** Knowledge of cervical cancer screening and HPV vaccination.

Question	Univariate Analysis			Multivariate Analysis		
	OR *	95%CI	*p*-Value ^†^	OR *	95%CI	*p*-Value ^†^
Necessity of regular cancer screening	1.29	(1.08–1.55)	0.005	0.96	(0.78–1.19)	0.695
Necessity of cancer screening from the age of 20	1.07	(0.89–1.30)	0.464	-	-	-
Cervical cancer negatively affects pregnancy	1.33	(1.11–1.60)	0.002	1.08	(0.87–1.34)	0.504
Cervical cancer may cause death	1.14	(0.97–1.35)	0.112	-	-	-
The virus leading to cervical cancer infects during sexual intercourse	1.19	(1.00–1.42)	0.048	0.872	(0.71–1.07)	0.19
The virus leading to cervical cancer is HPV	1.21	(0.99–1.47)	0.064	-	-	-
HPV vaccination is effective for cervical cancer prevention	2.03	(1.67–2.49)	<0.001	2.15	(1.69–2.74)	<0.001

* Logistic regression analysis; ^†^ Chi-squared test.

**Table 3:** Sexual activity and behavior.

	Vaccinated (*n* = 414)	Unvaccinated (*n* = 414)	*p*-Value
**Age at sexual debut ^1^ (mean ± SD)**	16.6 ± 2.2	15.8 ± 2.4	0.008 ^†^
**Number of previous sexual partners ^1^ (mean ± SD)**	4.0 ± 6.2	7.0 ± 12.4	0.016 ^‡^
**Experience of meeting a men via the Internet**			
Yes	98 (23.7%)	134 (32.4%)	0.003 *
No	314 (75.8%)	269 (65.0%)	
Unanswered	2 (0.5%)	11 (2.7%)	
**Condom use ^2^**			
Every time	80 (62.0%)	56 (47.5%)	0.046 ^§^
Frequent	28 (21.7%)	30 (25.4%)	
Occasional	9 (7.0%)	17 (14.4%)	
Seldom	2 (1.6%)	9 (7.6%)	
Never	8 (6.2%)	3 (2.5%)	
Unanswered	2 (1.6%)	3 (2.5%)	
**Experience of pregnancy ^2^**			
Yes	6 (4.7%)	14 (11.9%)	0.036 *
No	123 (95.3%)	99 (83.9%)	
Unanswered	0 (0.0%)	5 (3.4%)	
**Past history of sexually transmitted infections ^1^**			
Yes	10 (7.4%)	14 (9.5%)	0.72 *
No	124 (91.9%)	132 (89.2%)	
Unanswered	1 (0.7%)	2 (1.4%)	

^1^ experienced women (*n* = 283); ^2^ unmarried and experienced women (*n* = 247); * Chi-square test (exclude unanswered); ^†^ *t*-test; ^‡^ Mann-Whitney test.; ^§^ Chi-squared test (““every time” and “frequent”“ vs. ”“occasional”, “seldom” and “never””, exclude unanswered).

**Table 4:** Anxiety and preventive action against cervical cancer.

Question	Vaccinated (*n* = 414 )	Unvaccinated (*n* = 414 )	*p*-Value
**Are you afraid you might get cervical cancer?**			
terribly	139 (33.6%)	125 (30.2%)	0.48 *
yes	171 (41.3%)	175 (42.3%)	
no	85 (20.5%)	86 (20.8%)	
unknown	19 (4.6%)	28 (6.8%)	
**Are you afraid you might get sexually transmitted diseases?**			
terribly	136 (32.9%)	107 (25.8%)	0.15 *
yes	135 (32.6%)	143 (34.5%)	
no	143 (34.5%)	164 (39.6%)	
**Are you willing to have cancer screening regularly?**			
definitely	17 (4.1%)	23 (5.6%)	0.62 ^†^
probably	250 (60.4%)	234 (56.5%)	
no	85 (20.5%)	91 (22.0%)	
unknown	62 (15.0%)	66 (15.9%)	
**Vaccination is better way if vaccination can prevent cancer**			
agree	301 (72.7%)	223 (53.9%)	0.001 ^‡^
dysagree	33 (8.0%)	54 (13.0%)	
unknown	80 (19.3%)	137 (33.1%)	

* Chi-squared test ("terribly” and “yes”“ vs. “no”); ^†^ *t*-test (exclude unknown); ^‡^ Chi-squared test (exclude unknown).

## Data Availability

The data presented in this study are available on request from the corresponding author. The data are not publicly available due to restriction of privacy.

## Supplementary Materials

The following are available online at https://www.mdpi.com/2076-393X/9/2/87/s1, Table S1: Knowledge of cervical cancer screening and HPV vaccination.
