# Supplementary material for: Internet Survey of Awareness and Behavior Related to HPV Vaccination in Japan
Source: Vaccines (Basel). 2021 Jan 25;9(2):87. doi: 10.3390/vaccines9020087 (PMC7910902; doi:10.3390/vaccines9020087)
Supplement: Supplementary file 1 [file vaccines-09-00087-s001.pdf]

Table S1. Knowledge of cervical cancer screening and HPV vaccination.

Question	Vaccinated (*n* = 414)	Unvaccinated (*n* = 414)
**Necessity of regular cancer screening**		
savvy	92 (22.2%)	63 (15.2%)
vague	127 (30.7%)	122 (29.5%)
none	195 (47.1%)	229 (55.3%)
**Necessity of cancer screening from the age of 20**		
savvy	61 (14.7%)	46 (11.1%)
vague	77 (18.6%)	92 (22.2%)
none	276 (66.7%)	276 (66.7%)
**Cervical cancer negatively affects pregnancy**		
savvy	228 (55.1%)	192 (46.4%)
vague	130 (31.4%)	135 (32.6%)
none	56 (13.5%)	87 (21.0%)
**Cervical cancer may cause death**		
savvy	145 (35.0%)	123 (29.7%)
vague	124 (30.0%)	130 (31.4%)
none	145 (35.0%)	161 (38.9%)
**The virus leading to cervical cancer infects during sexual intercourse**		
savvy	92 (22.2%)	65 (15.7%)
vague	81 (19.6%)	90 (21.7%)
none	241 (58.2%)	259 (62.6%)
**The virus leading to cervical cancer is HPV**		
savvy	56 (13.5%)	43 (10.4%)
vague	72 (17.4%)	61 (14.7%)
none	286 (69.1%)	310 (74.9%)
**HPV vaccination is effective for cervical cancer prevention**		
savvy	89 (21.5%)	37 (8.9%)
vague	168 (40.6%)	123 (29.7%)
none	157 (37.9%)	254 (61.4%)
